# The importance of microenvironment: the role of CCL8 in metastasis formation of melanoma

**DOI:** 10.18632/oncotarget.5059

**Published:** 2015-07-31

**Authors:** Tamás Barbai, Zsuzsanna Fejős, Laszlo G. Puskas, József Tímár, Erzsébet Rásó

**Affiliations:** ^1^ 2nd Department of Pathology, Semmelweis University, Budapest, Hungary; ^2^ Avidin Ltd., Szeged, Hungary; ^3^ MTA-SE Tumor Progression Research Group, Budapest, Hungary

**Keywords:** melanoma metastasis, microenvironment, CCL8, miR146a

## Abstract

We have attempted to characterize the changes occurring on the host side during the progression of human melanoma. To investigate the role of tumor microenvironment, we set up such an animal model, which was able to isolate the host related factors playing central role in metastasis formation. One of these ‘factors’, CCL12, was consequently selected and its behavior was examined alongside its human homologue (CCL8). In our animal model, metastasis forming primary melanoma in the host exhibited increased level of CCL12 mRNA expression. In clinical samples, when examining the tumor and the host together, the cumulative (tumor and host) CCL8 expression was lower in the group in which human primary melanoma formed lung metastasis compared to non-metastatic primary tumors. We could not detect significant difference in CCL8 receptor (CCR1) expression between the two groups. Increased migration of the examined tumor cell lines was observed when CCL8 was applied as a chemoattractant. The tumor cells and their interactions can be influenced the expression of CCL8 by dermal fibroblasts, as a significant change in the metastatic microenvironment. Furthermore, we examined changes in miRNA profile resulted by CCL8 and miR146a appears to be a promising prognostic marker for following this process.

## INTRODUCTION

Clinicians face the most difficult of treatment plans when it comes to metastatic melanoma. Surgical resection of the primary tumor and resection/treatment of the regional lymph nodes in stage II and resectable stage III melanoma could lead to a disease free status. The treatment of unresectable stage II, stage IV and recurrent melanoma is not quite as straightforward with numerous treatment options. The response rate to conventional chemotherapy (DTIC, Temozolomide) is only 10-15% with even lower occurrence of complete remission. Therefore the fact that melanoma is a highly immunogenic tumor raised high hopes for immune therapies. Most of these are immune checkpoint inhibitors, such as anti-CTLA-4, anti-PD-1. The discovery and introduction of signal transduction inhibitors, BRAF inhibitors (for tumor harboring V600E mutation), MEK inhibitors targeting MEK1 and MEK2, both of which are downstream in the BRAF signal transduction pathway, multikinase inhibitors and KIT inhibitors have brought a revolutionary change in melanoma therapy. Although the response rate significantly increased, there was no dramatic change in overall survival, despite the expectations. It is therefore of utmost importance that we understand the mechanism of progression and metastasis formation of melanoma [1].

The basis of metastasis research is examining each step of the cascade of tumor progression. Stages in this process are separated into two aspects: tumor and host related factors, with the distinction between the two sometimes blurred. Interaction between tumor cells and their microenvironment does not only provide opportunity for tumor growth, but can also lead to the selection of a metastatic population of tumor cells [2]. This interaction can form the metastatic niche, by providing an angiogenic phenotype [3], extracellular matrix rearrangements via production and storage of chemokines and growth factors [4, 5, 6], changes in migration potential [7], and will lead to tumor formation of the selected cells due to stromal cell activation [8]. The primary stromal components of importance are the fibroblasts, which are reprogrammed as tumor associated fibroblasts by tumor cells, and provide part of the molecular environment for the primary tumor [9]. Surprisingly, it is this environment that provides the premetastatic and metastatic niche at the sites of distant metastases and promotes colonization by the selected tumor cell clones [10, 11]. Several genes have been identified as participants in the activation of the metastatic cascade, however the complete spectrum of genes has yet to be revealed. [12, 13, 14].

Chemokines are small molecular weight proteins which can bind specific G protein coupled cell surface receptors [15]. They play a key role in many areas, such as migration, inflammation, immune response and tumor growth. The classification of chemokines is based on the pattern of the cysteine residue containing protein C terminus which can be divided into four major groups: CXC, CX3C, CC and C, where C is cysteine and X denotes any other amino acid. Functionally, there are homeostatic and inflammatory cytokines [15]. The nomenclature of cytokines is not uniform, special attention should therefore be paid to make sure that a certain human chemokine molecule is paired and examined with its correct, homologous mouse counterpart. [16]. Several members of the tumor-host system have been proven to take part in tumor progression, such as tumor growth, migration, angiogenesis and EMT [17, 18]. The metastatic cascade is the step by step process, which represents the route by which tumor cells can travel from the primary tumor through the blood and lymph systems [19]. The occurrence of metastases shows a certain pattern, indicating that it might not be a random set of events. Physiologically, chemokines are involved in the management of leukocyte migration and by a similar method they promote organ specific tumor metastasis formation. In the most studied, CXCR4/CXCL12 system, tumors overexpressing CXCR4 had high metastatic potential to CXCL12 expressing organs, i.e. mostly lymph nodes [20]. This correlation can be observed in multiple tumor types (eg. breast [21], colorectal [22], prostate [23] and melanoma [24] tumors). Likewise, clear correlation was shown between CCR7 expression and lymph node metastasis formation via lymphatic vessels in case of head and neck cancers, NSCLC and melanoma [25, 26]. Tumors showing de novo expression of CCR7 in a murine isograft system [27] (B16F10 melanoma bearing animals) showed lymph node metastases, whereas lung metastases were formed in the absence of CCR7 expression [28]. The same connection was demonstrated in human clinical samples. A similar relationship was observed between lymphogenic metastasis and CXCR3 expression. Namely, in a B16F10 melanoma mouse model, the loss of CXCR3 expression was demonstrated to reduce the incidence of lymph node metastasis by 15% [29]. The importance of the chemokine system in melanoma metastasis formation is further supported by a study in which high levels of CCR9 in the primary tumor promoted metastasis formation in the small intestine [30], whereas increased CCR10 level induced the appearance of skin metastases [31]. The CC chemokine, CCL8 (MCP2) molecule was investigated during inflammatory response for its monocyte and T-lymphocyte attractant properties. The cell surface receptors of CCL8 are CCR1, CCR2 and CCR5, all of which belong to the CC chemokine receptor family [32]. Examining the host-derived factors affecting the behavior of human melanoma in our experimental models, we have demonstrated CCL8 to have a regulatory role during melanoma progression.

## RESULTS

**Figure 1:**
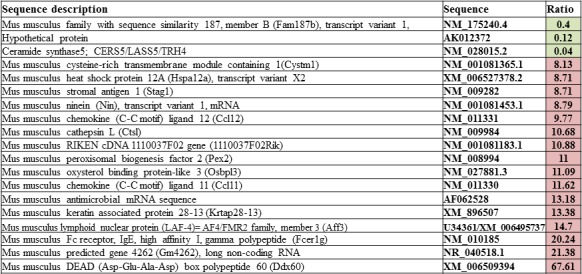
Gene sets from microarray experiment Single cell suspension of HT168M1 human melanoma cell line was semiorthotopically implanted into newborn SCID mice. The RNA expression pattern of this, metastatic version of the primary tumor was investigated via Agilent Mouse Oligo Microarray Platform. The stromal expression changes were compared to healthy, age matched controls and the nineteen genes showing the largest expressional changes were selected for further validation.

Single cell suspension of the human melanoma cell line, HT168M1 was orthotopically implanted into adult and newborn SCID mice simultaneously. There was primary tumor growth in both hosts, but metastases only formed in the newborn host. The experiment was terminated after 30 days and RNA isolated from the metastatic primary tumor (newborn host). The RNA expression pattern of the metastatic host was investigated by Agilent Mouse Oligo Microarray Platform containing 20,000 genes. The stromal expression changes were compared with tissue derived from corresponding area of tumor implantation of healthy, age-matched controls. The nineteen genes showing the largest expression changes (Figure 1) were then chosen and their expression further examined on samples from three genetically different human melanoma cell lines (HT168M1, WM983B, HT199) implanted into the same metastatic / non-metastatic model. The species specificity of the primers for every examined gene was checked by using them on human and mouse tumor cell lines. This was necessary to ensure that the expression changes were strictly mouse-derived stroma related, therefore only those primers giving clear products in the target species (mouse), but not in human, were validated. PCR products were identified by direct sequencing. Nine out of 19 genes were suitable to be used as host specific markers: Fam 187b, Lass5, DEAD box 60 polypeptide, Gm4262, CCL11, Pex2, Cathepsin L, CCL12 and Ninein. Total RNA was then isolated from the implanted *in vitro* cultures and *in vivo* subcutaneous primary tumors of all three melanoma cell lines, and the relative expression of target genes was determined by real time PCR. To assess the role of these genes in providing a microenvironment prone to select a more metastatically potent subset of cells, we compared the relative expression pattern of these genes in the non-metastatic and metastatic version of each of the three melanoma cell lines growing in adult and newborn hosts respectively.

**Figure 2:**
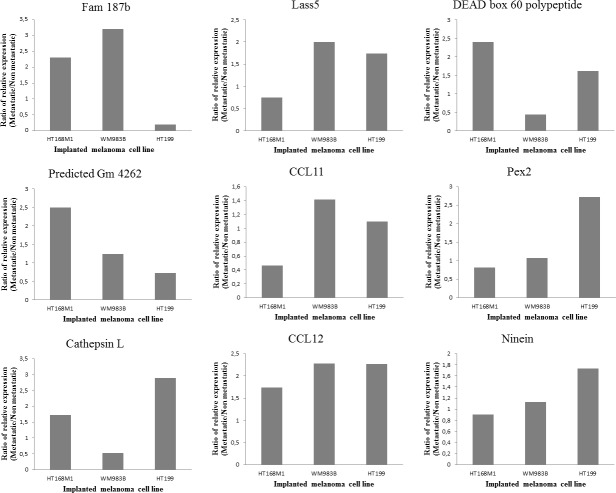
Validation of microarray data Three different human melanoma cell lines (HT199, HT168M1, WM983B) were semiorthotopically implanted at the same time into newborn (metastatic version) and adult (non-metastatic version) SCID mice. Lung metastases were detected only in the newborn host. Nine out of the previously selected 19 genes were suitable for host specific PCR. Their relative expression was measured in xenotransplanted metastatic and non-metastatic animal models. In every case, changes in expression of the target gene in the host of the metastatic and non-metastatic version of the primary tumor were compared with the expression level of the beta microglobulin housekeeping gene. The results were displayed as the ratio of expression levels in the two hosts, with the changes considered significant if the ratio was higher than 1.5-fold.

**Figure 3:**
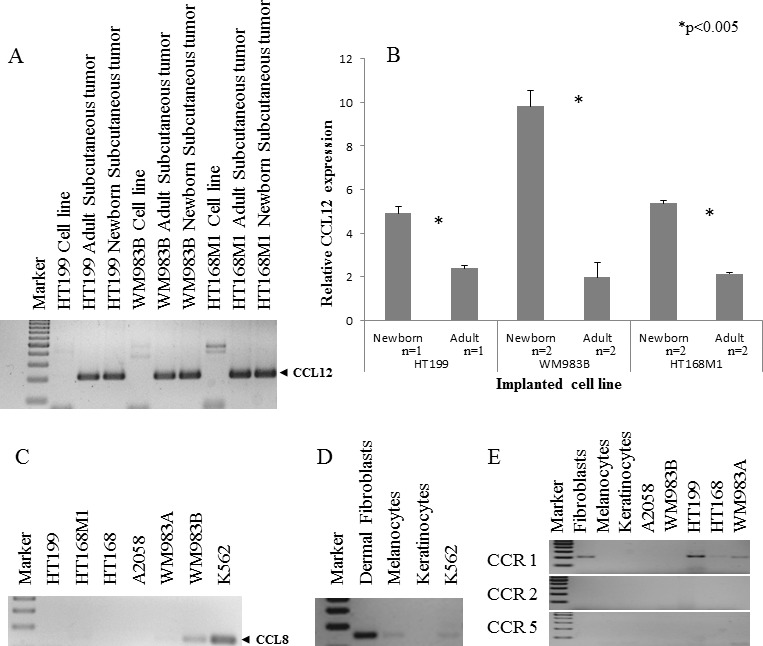
Qualitative and quantitative expression of CCL12 and CCL8 **A.** The qualitative CCL12 pattern showed that the host specific PCR was appropriate to discriminate between the implanted human melanoma cell line and the experimental tumor samples. **B.** Three human melanoma cell lines (HT199, WM983B, HT168M1) were implanted into newborn and adult SCID mice. A more than 1.5 fold change of CCL12 relative expression was detected between the primary subcutaneous tumor from the newborn (metastatic model) and adult (non-metastatic model) animals at RNA level. Data presented are mean values ± SD. **C.** CCL8 expression was investigated in six different human melanoma cell lines and was detected in only one of them (WM983B). **D.** The expression of CCL8 (human homologue of CCL12) was demonstrated in non-tumoral (dermal fibroblast, melanocyte) cells. The positive control was the ubiquitously CCL8 expressing K562 leukemia cell line. **E.** The expression of CCL8 receptors (CCR1, CCR2 and CCR5) was analyzed in normal cells (dermal fibroblasts, melanocytes, keratinocytes) and tumor cell lines (HT199, A2058, WM983A, WM983B, HT168). CCR1 expression was only detected in fibroblasts and three different melanoma cell lines (HT199, HT168, WM983A).

The expression threshold used was 1.5 fold or higher in at least two cell lines. Five of the nine genes fulfilled these requirements, namely Fam 187b, Lass5, DEAD box 60 polypeptide, Cathepsin L and CCL12 (Figure 2). Unfortunately, any PCR-based technique would struggle to differentiate between host and tumor-derived expression of genes due to their genetic identity (Figure 3A). Therefore, human homologues of the five genes were analyzed in our human melanoma cell lines. There was only one gene, CCL12 and its human homologue CCL8, which was expressed by only one human melanoma cell line (WM983B, which was derived from a melanoma metastasis) and therefore was proved to be suitable for further analysis. An almost two-fold difference was observed in the relative expression level of CCL12 between metastatic and non-metastatic model in all tested cell lines (Figure 3B). This result ‚nominated‘ this gene to operate in the selection of metastatic tumor cells.

We then examined the expression of CCL8 in five, genetically different human melanoma cell lines (HT199, HT168M1, WM983A, A2058, WM983B) and found that it was expressed by only one of them (WM983B) (Figure 3C). Additionally, we demonstrated CCL8 expression in *in vitro* cell cultures of human dermal fibroblasts and melanocytes (Figure 3D). In order to find out which cell types are the potential targets of CCL8, the presence of CCL8 sensitive chemokine receptors was analyzed in the tumorous and several normal constituents of the tumor-host system. Specifically, we examined the expressions of CCR1, CCR2 and CCR5 (Figure 3E). Expression of CCR1 was detected in HT199, HT168 and WM983A tumor cell lines. Only dermal fibroblasts expressed CCR1 from the host side.

**Figure 4:**
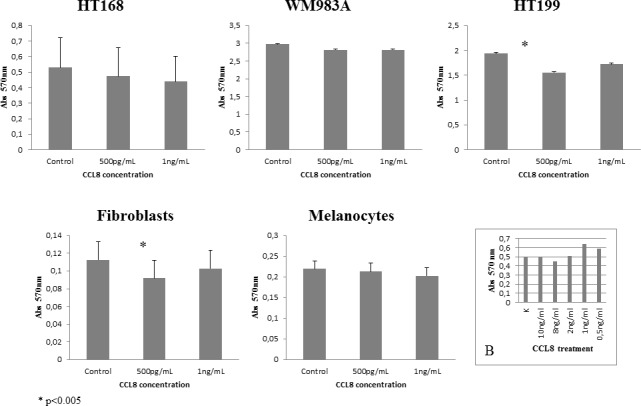
Effect of CCL8 on cell viability The effect of CCL8 on cell viability was detected in the presence of two different concentrations (500 pg/ml and 1 ng/ml) of human recombinant CCL8 and was compared to untreated control, after 12 hours of treatment. The viability changes were measured by MTT test, where absorbance was detected on 570 nm proportionate to the number of living cells. Every tumor cell line and fibroblasts carried the CCL8 receptor, CCR1. Significant differences were observed in HT199 (tumor cell line) and dermal fibroblasts (host cell) at the concentration of 500 pg/ml, where viability was reduced in both cases. Data presented are mean values ± SD of absorbance obtained from 18 parallel samples in one representative experiment. *p < 0.005. **B.** Concentration range of CCL8 treatment on HT168 cell line.

After identification of the chemokine/receptor pattern of both sides of the tumor-host system, our next step was to map its functional effects. First of all, the effect of CCL8 on dermal fibroblasts, melanocytes and CCR1-expressing tumor cell lines was examined, using MTT-test. The effect of CCL8 on viability was compared to untreated control, using two different concentrations of CCL8, after 12 hours of treatment. To determine the optimal concentration range of the treatment we tested the viability of HT168M1 human melanoma cells. Based on the viability test the applied concentration was 0.5-10 ng/ml (Figure 4B).

Significant difference was observed in HT199 of the tumor cells lines and in dermal fibroblasts of host cells in both cases at the concentration of 500 pg/ml, which reduced viability (Figure 4).

**Figure 5:**
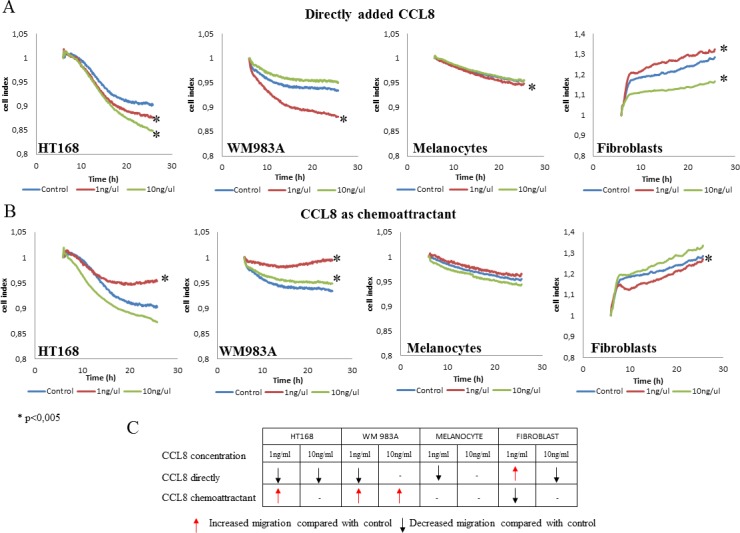
Effect of CCL8 on migration **A.** Cells were treated with recombinant human CCL8, added directly to the cells in two different concentrations. Graphs represent the migration activity of cells 5 hours after treatment related to18 hours of data collection. In case of melanocytes the control line and the line of 10 ng/ul runs together. **B.** CCL8 was applied as a chemoattractant in two different concentrations, where cells migrated towards the chemokine source through a transwell membrane. Graphs represent the migration activity of cells 5 hours after treatment related to 18 hours of data collection. Data presented are cell index. *p < 0.005. **C.** Summarized results represent that directly added CCL8 inhibited tumor cell migration while as a chemoattractant it increased cell motility. The migration of non-tumor cells was either not altered, or was inhibited by CCL8 as a chemoattractant (fibroblasts in low concentration).

The effect of CCL8 on migration, one of the key steps of the metastatic cascade, was measured with xCELLigence-system on CIM (Cell Invasion Migration) plate, semiquantitatively. Based on the detection of impedance by microelectrodes, the migration activity of the untreated control culture was compared with that of the treated cells. CCL8 was applied in two different ways on cell cultures: either added directly to the culture or as a chemoattractant. In both cases the administered concentrations were 1 and 10 ng/ml. Analyses started 5 hours after treatment following which data were collected every 5 minutes for a duration of 18 hours. A summary of the results is presented in the table inserted in Figure 5C. According to our observations, the migration of tumor cells was inhibited by CCL8 when applied directly and it was increased when added as chemoattractant. On the other hand, CCL8 as a chemoattractant either did not alter the migration of non-tumor cells or inhibited them (fibroblasts in low concentration) (Figure 5). When applied directly, CCL8 inhibited migration of both melanocytes and fibroblasts, while in lower concentration it significantly increased migration activity of fibroblasts.

Since the expression pattern of CCL8 was identified in the melanoma cell lines and non-tumoral cells (fibroblasts, melanocyte, keratinocyte) as well as human metastatic and non-metastatic melanoma samples in our metastatic animal model, we raised the question whether there was any correlation between chemokine expression and metastasis formation of human primary melanomas.

**Figure 6:**
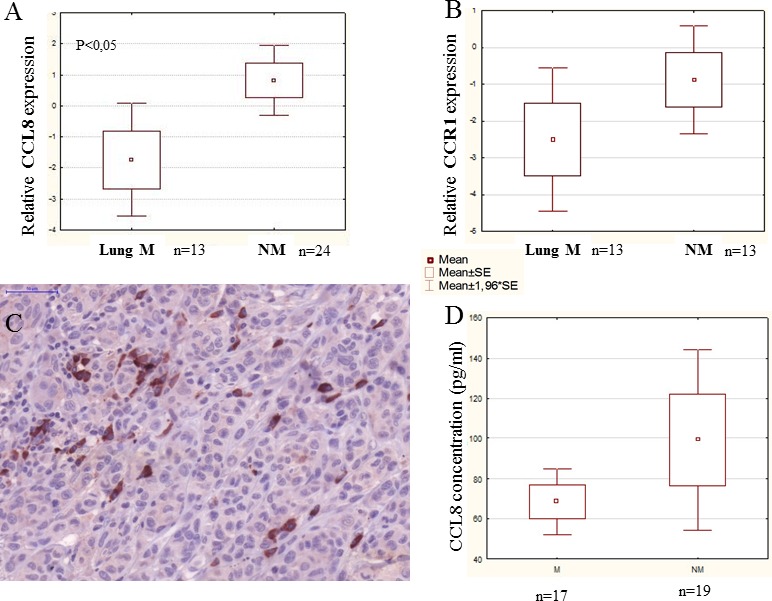
CCL8 expression in human primary melanoma samples Human melanoma samples were divided into two groups: non-metastatic primary tumor (NM - no metastases detected during the five year follow-up) and metastatic primary tumor (M - the patients developed metastases within five years). Patients of the metastatic group had distant organ metastases in the lung, liver, brain, bones, skin and lymph nodes. **A.** Relative expression of CCL8 in clinical samples was measured by real time PCR and significant differences were detected between non-metastatic and lung metastatic primary human melanomas, the latter expressing lower quantities of CCL8. **B.** There was no difference in CCR1 expression level between the two groups (lung metastatic vs. non-metastatic). **C.** Heterogeneous expression of CCL8 protein was detected in the human melanoma primary tumor samples by immunohistochemistry (magnification: 20x). **D.** Systemic appearance of CCL8 in serum of melanoma patients was analyzed by ELISA kit. Thirty-six samples were analyzed and CCL8 protein was detected. Differences found between metastatic and non-metastatic samples were not stasistically significant, because deviation of the datas.

Human surgical primary melanoma samples were divided into two groups: non-metastatic (NM) primary tumor - patients with no metastasis detected during a follow-up period of five years and metastatic (M) primary tumor - patients developing metastasis within five years. Data were retrospectively collected to include sex, age, tumor phenotype (pigmentation), tumor site, histological type, Clark level and Breslow thickness (Table 1A). Patients of the metastatic group had distant organ metastases (single or multiple) in the lung, liver, brain, bones, skin and lymph nodes (Table 1B). Total RNA was then isolated and the CCL8 expression determined in each sample, qualitatively in the first instance. Subsequently, the relative quantitative expression of CCL8 was also measured by real time PCR, comparing the CCL8 expression level with human beta actin expression (relative CCL8 expression). A total of 57 clinical primary melanoma samples were analyzed and no statistical difference was found between the metastatic and non-metastatic group. Metastatic primary tumor samples were then further divided into 6 subgroups (patients with lung, liver, skin, bone, brain and lymph node metastases) based on the target organ of the metastases. Because of patients with multiple metastases in different locations the number of samples proved to be sufficient for our study only when we examined primary tumors forming lung metastases. When re-analyzing the data considering this subgroup, we found a significant difference between non-metastatic primary human melanoma and primary melanoma with lung metastases, the latter group expressing lower levels of CCL8 (Figure 6A).

**Table 1:**
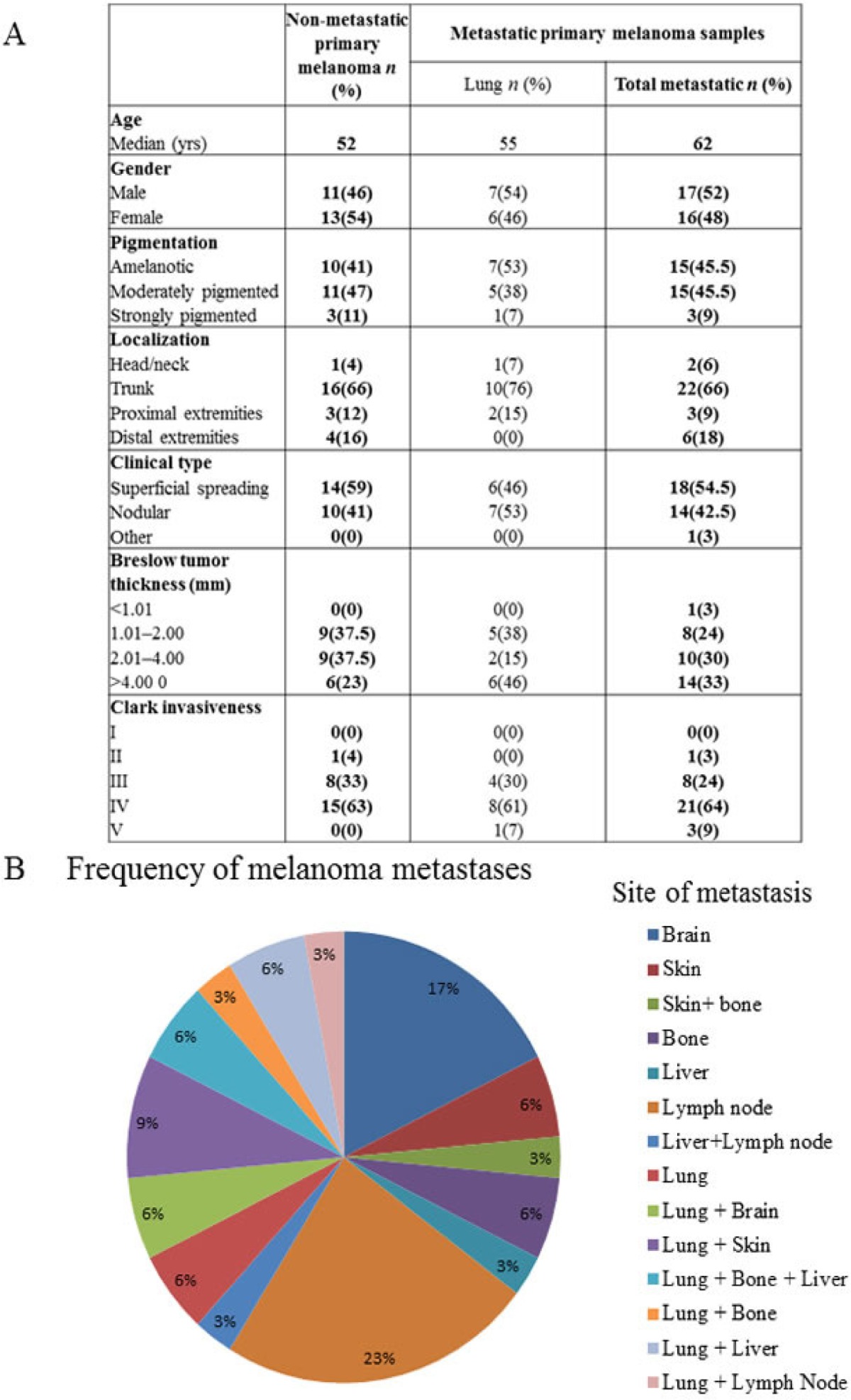
Clinical characteristics of the patient groups

The expression of the CCL8 receptor, CCR1, was analyzed on the same samples, but no difference was observable between the analyzed non-metastatic primary human melanoma and primary melanoma with lung metastases groups (Figure 6B).

It was not possible to separately examine CCL8 mRNA expression of the stromal components in our surgical samples, therefore we used immunohistochemistry to reveal the localization and expression of CCL8. Heterogeneous expression of this protein was detected primarily in the stromal cells, and in the tumor as well, though to a lesser extent (Figure 6C). Systemic appearance of CCL8 in the serum of patients with melanoma was analyzed by ELISA kit, with recombinant CCL8 used to set the standard curve. During measurement, because of the detection limit, CCL8 concentrations lower than 60 pg/ml were considered to be zero. Altogether, 36 serum (17 metastatic and 19 non-metastatic) samples of patients with melanoma were analyzed and CCL8 protein concentration was higher than 60 pg/ml in 15(7 metastatic and 8 non-metastatic) of the analyzed serum samples. The level of CCL8 protein was found to be greater in non-metastatic group of patients, however because of low sample number and broad range of standard deviation it did not prove to be statisically significant (Figure 6D).

**Figure 7:**
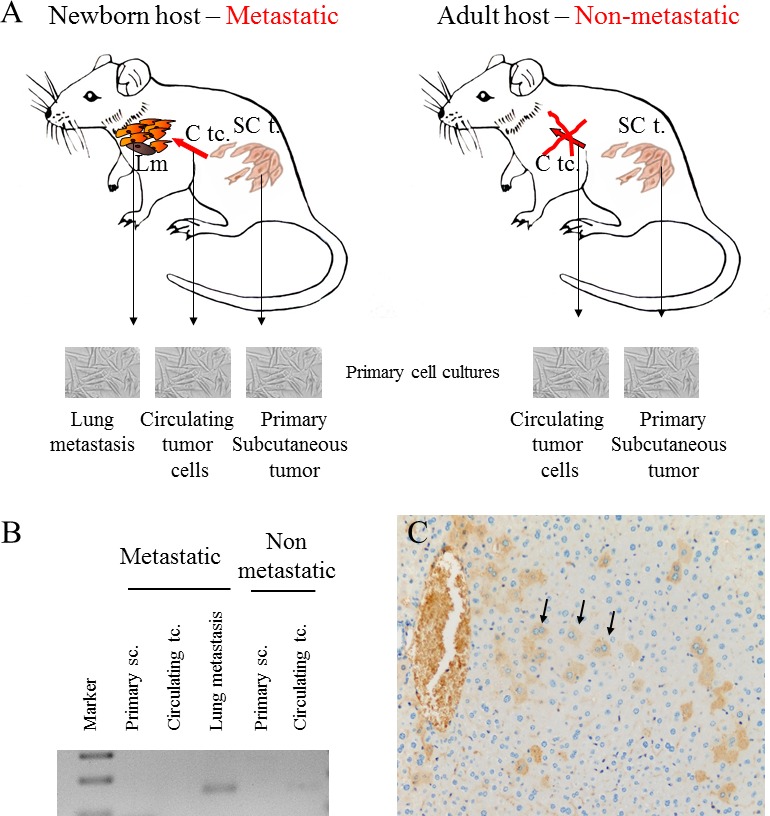
CCL8 expression of tumor cells from different stages of the metastatic cascade **A.** Animal model of human melanoma progression. Primary tumors from adult and newborn hosts (SC t.: subcutaneously implanted tumor) were removed along with lung metastases (Lm), which were formed only in newborn mice. Primary cell cultures were created from all of the above tumors and from the circulating tumor cells (C tc.) of newborn and adult mice. **B.** CCL8 expression was qualitatively detected after mRNA isolation from the metastases of the metastatic system (newborn) and from the circulating tumor cells of the non-metastatic system (adult). **C.** CCL8 expressing tumor cell clones were visualized at protein level in the metastatic (newborn) primary xenograft tumor by immunohistochemistry (magnification: 20x).

**Figure 8:**
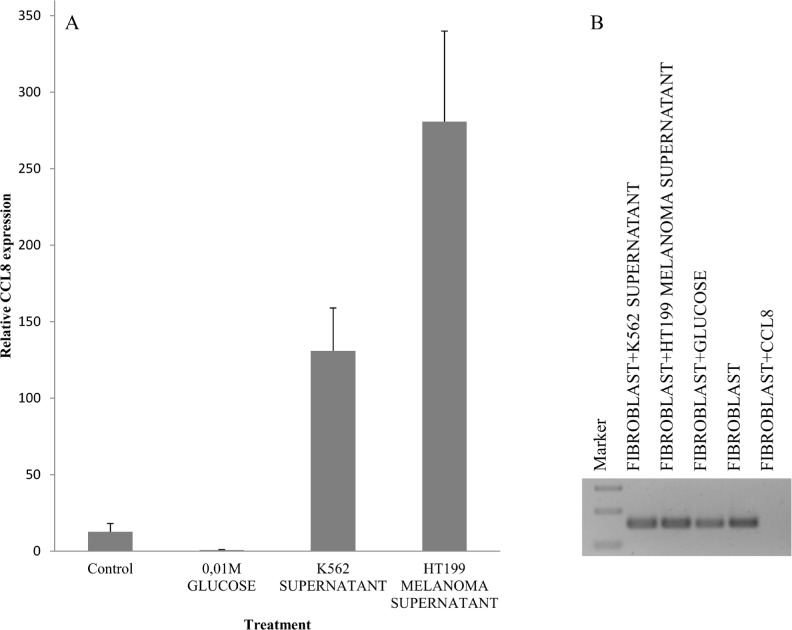
Simulation of host-tumor cooperation Human dermal fibroblast cells were treated with HT199 melanoma and K562 leukemia cell-free supernatants (to simulate the effect of secreted tumoral factors) and pure, recombinant human CCL8. We used untreated cells and samples treated with 0.01M glucose solution as controls. CCL8 relative expression was measured by real time PCR. **A.** Increase in CCL8 expression was observed after melanoma and leukemia supernatant treatment. **B.** In contrast, pure CCL8 treatment terminated the continuous expression of CCL8.

Our experimental animal model offered good possibility to examine tumor cell populations derived from different stages of the metastatic cascade, free of stromal components. Namely, cell cultures were created from metastatic and non-metastatic primary tumors, circulating tumor cells as well as lung metastases, and CCL8 expression was measured qualitatively. CCL8 mRNA expression was detected in the lung metastases of the metastatic system (newborn model) and in circulating tumor cells of the non-metastatic system (adult model) (Figure 7) We could not prove the presence of CCL8 mRNA in cell culture derived from the primary tumor, although by analyzing the whole subcutaneous tumor tissue CCL8 was detectable by immunohistochemistry in a small group of tumor cells (Figure 7C). To further investigate the molecular cooperation between host and tumor, we treated human dermal fibroblasts with the supernatant of HT199 melanoma and K562 leukemia cell cultures as well as 'pure' CCL8. Untreated cells and glucose solution were used as control. Dramatic increase of CCL8 expression was observed after melanoma and leukemia supernatant treatment, but in contrast 'pure' CCL8 treatment terminated the continuous expression of CCL8 (Figure 8).

**Figure 9:**
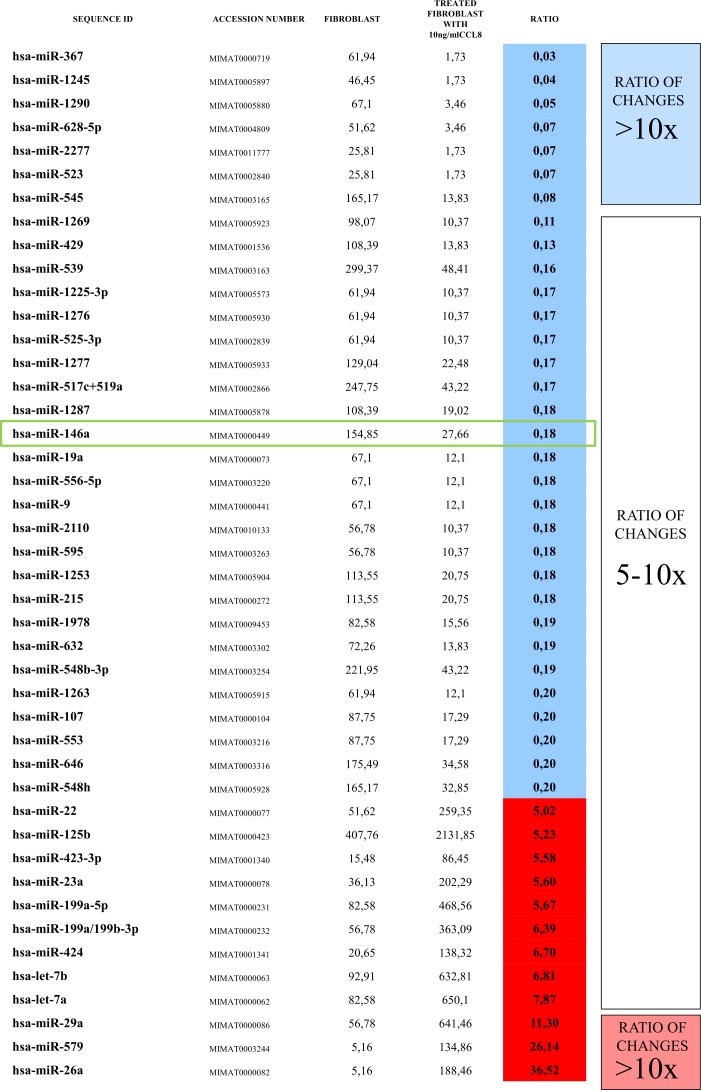
CCL8 specific miRNA pattern Human dermal fibroblasts were treated for 12 hours with human recombinant CCL8. MicroRNA quantitative profile was analyzed by nCounter miRNA Expression Assay Kit from NanoString. The changes were compared to miRNA levels measured in untreated control fibroblasts. MicroRNAs which showed fivefold or higher difference between the two groups were accepted, thus we created a CCL8 specific microRNA pattern. miR146a, which was localized in the middle range of our list, has already been described to participate in the regulation of CCL8, according to the literature [37].

**Figure 10:**
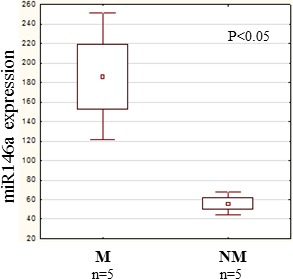
miR146a expression in human primary melanoma We investigated the quantitative expression of miR146a in randomly selected 5 metastatic and 5 non-metastatic primary melanomas. A significantly higher miR146a expression level was measured in the metastatic group (independent of the localization of the metastasis) than in the non-metastatic group. Data presented are mean values ± SE.

The rapid effect of CCL8 on the gene expression of dermal fibroblasts suggests that microRNAs might be involved. In the two groups (CCL8 treated and control) the quantitative change of 800 human microRNAs was measured. We created a CCL8 specific microRNA pattern by including microRNAs showing fivefold or higher differences between the two groups. One of the microRNAs, miR146a, was also found in the middle range of our list (Figure 9), therefore playing role in the regulation of CCL8 in accordance with the literature [37]. miR146a level was measured in randomly selected five samples each of the metastatic and non-metastatic primary tumors of human melanoma patients, using nCounter assay. A statistically significant, higher miR146a expression level was found in the metastatic group (independent of metastasis localization) as compared with the non-metastatic group (Figure 10).

## DISCUSSION

The microenvironment, as one of the key components regulating tumor behavior, can take part in the destruction of small tumors, as well as sometimes support tumor growth and migration [34]. The combination of these two effects provides the selection pressure generating a specific tumor cell population: the metastatic tumor cell population.

The metastatic phenotype (genotype) is the feature of tumor cells which are able to detach and leave the primary tumor, settle and form a new colony in distant organs [35]. Undoubtedly, this geno/phenotype develops as the result of a selection pressure, in which the host-derived microenvironment plays a key role. We attempted to identify the molecular markers of this tumor-host interaction and examine their role in metastasis formation in our experimental animal model system. We have identified differences in the expression pattern of the examined genes in the metastatic and non-metastatic version of the same tumor type. However, it is yet to be decided if a specific pattern is homogenous throughout the entire tumor, or it is present only in a small subset of cells which are able to enter the metastatic cascade. If the latter is true, it is possible that beyond a certain threshold, a high enough quantity of this metastatically more potent subset can have a significant effect on the whole of the tumor, and can therefore be used as a prognostic marker of metastasis formation. CCL12 was one of the genes we identified as a possible host-related factor which might be involved in supporting a metastatic phenotype. This gene showed a more than 1.5-fold increased expression in the metastatic version of primary tumors, when compared to their non-metastatic counterparts in all three of our xenograft animal models. We also showed that, with the exception of one (WM983B), all the examined human melanoma cell lines lacked expression of CCL8, the human homologue of CCL12. To identify the cells expressing CCL8 at the site of implantation, we analyzed the expression of this protein in dermal fibroblasts, melanocytes and keratinocytes. Among these cells, fibroblasts and melanocytes expressed the chemokine CCL8. However, it is not enough to simply identify the expression of these chemokines. One must aim to discover their interactions by which they affect the complex process of metastasis formation. The effect of CCL8 is mediated by special chemokine receptors CCR1 and CCR5. CCR1 expression was identified in fibroblasts and three of the five melanoma cell lines examined. None of the cell lines in our study showed CCR5 expression. Another aspect of the local effect of chemokines on cells is the way they affect viability and migration. The viability of one of the 3 studied melanoma cell lines and dermal fibroblasts was similarly influenced by CCL8 statistically, although this might not be a biologically relevant effect. In melanoma cell lines CCL8 increased the migration potential when added as a chemoattractant, while it did not alter the motility of host cells. In conclusion, CCL8 may enhance the ability of metastasis formation in melanomas as a chemoattractant.

Our xenotransplantation animal model gave opportunity to study the behavior of tumor cells originating from various steps of the metastatic cascade. Namely, tumor cells from the primary tumor, circulating tumor cells and cells from lung metastasis were investigated *in vitro*. Our results showed that cell cultures from the primary tumor did not express the chemokine while cells isolated from the circulation and metastases both expressed CCL8. However, it is difficult to draw conclusion based on these findings, since circulating tumor cells represent a so called intermediate state, which merely carries the possibility and not a guarantee of metastasis formation.

Since we revealed a quantitative expression difference of CCL12 between host cells of metastatic and non-metastatic xenograft primary tumors, we studied whether this expression difference could be detected in human clinical samples as well and whether it could possibly be used as prognostic marker to indicate tumor progression.

We analyzed clinical samples of patients suffering from melanoma We divided these samples into two groups: non-metastatic primary tumor (metastases did not occur during a five year follow-up) and metastatic primary tumor (metastases formed within five years). The quantity of CCL8 was measured by ELISA test in the serum of these patients. In some samples the chemokine was not present at all. Average CCL8 protein level was higher in the non-metastatic than in the metastatic group, but this difference was not statistically significant. Measuring the relative expression of CCL8 in human primary tumors, we did not find any distinction between the non-metastatic and metastatic groups. However, comparison between the chemokine expression of primary tumors from patients with and without lung metastases showed significant difference. The expression of the receptor (CCR1) mediating the effects of CCL8 was similar in the two primary tumor groups. There was no difference in expression of the receptor (CCR1) mediating the effects of CCL8 between the two primary tumor groups. In order to decide which cell types could be the source of CCL8 production (stromal cells or tumor cells), immunohistochemistry was performed on clinical samples. We found that both stromal and tumor cells expressed CCL8, but the number of positive cells were higher in stroma. The small number of cells synthetizing this chemokine suggests a local rather than an overall effect.

We then examined the effect of two cell line supernatants (K562, HT168M1) on the CCL8 expression of dermal fibroblasts *in vitro*. Significant increase was found shortly after the treatment, while addition of pure CCL8 resulted in direct and complete reduction. This phenomenon could be due to miRNAs, which exhibit a regulatory function [36]. To reveal which miRNAs are responsible, the expressions of 800 miRNAs were studied in dermal fibroblasts, with expression level changes found in many of these miRNAs after CCL8 treatment. One of the candidate miRNAs we identified was miR146a, which has already been described as having a regulatory effect on CCL8 expression [37]. In our study, the expression level of this miRNA showed a significant difference between patients with metastatic and non-metastatic primary melanoma.

Based on our results in xenotransplantation animal model and human clinical samples, we suggest that CCL8 and its mouse homologue play a role in the metastatic cascade. The CCL8 expression of dermal fibroblasts was increased by soluble factors, produced by the tumor itself. CCL8 in return enhances the migration activity of tumor cells as chemoattractant.

According to our results, pure CCL8 treatment decreases the CCL8 expression of fibroblasts. It is a snapshot of the delicate balance of the stimulating effect of the tumor and the inhibition of CCL8 itself that we see during molecular studies. The role of numerous chemokines has been proven in the metastasis formation of human melanoma. Our studies support the role of a new member of metastasis associated chemokines, CCL8, in the formation of lung metastases.

**Figure 11:**
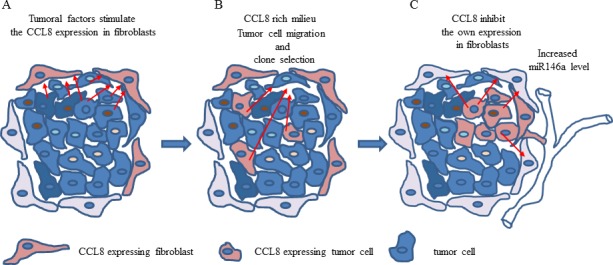
The hypothetical effect of CCL8 in host-tumor system **A.** Soluble factors produced by tumor cells stimulate the CCL8 expression of dermal fibroblasts in the stroma. **B.** The increasing concentration of CCL8 and its chemoattractant ability results in an increased migration of tumor cells to the stroma. It also takes part in the selection of potentially CCL8 expressing tumor cells harboring metastatic potential. **C.** In this case the increasing CCL8 concentration can regulate its own expression in dermal fibroblasts. The change in miR146a expression (overexpressed in dermal fibroblasts in this case) serves as an appropriate marker for this process.

The apparent contradictions between our data from the animal model and clinical samples can be explained by a few facts. In the animal model we were only able to detect the relative chemokine expression of host cells with host (mouse) specific methods in contrast to clinical samples in which the whole cumulated (tumor and stroma) expression level was measured in order to validate the prognostic value. In conclusion we created a hypothetic process: factors of tumoral origin stimulate the chemokine expression of cells in their surroundings, mostly in fibroblasts and this chemokine functions as a chemoattractant molecule. As a consequence of the tumor-host interaction, some of the tumor cells might be able to express CCL8. The local CCL8-rich environment could promote the selection of tumor cells with metastatic ability. The high CCL8 concentration inhibits the migration of tumor cells. *In vitro* studies showed that the exogenous CCL8 blocks the production of this protein in fibroblasts, thus if a CCL8 expressing population grows in the primary tumor, these cells could regulate the chemokine expression of tumor associated fibroblasts. Our data suggests that miR146a expression and CCL8 level are related. Therefore, miR146a could contribute to restoring the shifted balance, meaning that it is overexpressed in metastatic tumors. By contrast, in non-metastatic tumors low miR146a expression level is accompanied by relatively high CCL8 expression (Figure 11). However, this hypotesis should be supported by further analysis.

## MATERIALS AND METHODS

### Human xenograft model

Xenotransplantation was carried out by subcutaneous inoculation of freshly prepared cell suspension of human melanoma cell lines (HT199, HT168M1, WM983B, 5×10^6^ cells/50 μl/animal) in the left hind leg of adult and newborn animals. On the 30th day, the animals were sacrificed by bleeding under anesthesia. Primary *in vitro* cell cultures were formed from the primary tumor, circulating tumor cells and lung metastases of the same animal implanted as a newborn. Further, the primary tumor and circulating tumor cells from the adult animals were used to create cell cultures in a similar manner. This way the 'noise' of communication with the microenvironment, such as skin, blood, lung, was excluded. Total RNA was isolated from the cultures.

This study was carried out in strict accordance with the recommendations and was approved by the Semmelweis University Regional and Institutional Committee of Science and Research Ethics (TUKEB permit number: 83/2009).

### Tumor samples

Frozen samples of melanoma were stored in RNA later. Random sampling was applied throughout the experiments. Stratification of melanomas was based on the percentage of melanoma cells containing melanin. Melanomas were classified as amelanotic, moderately pigmented and strongly pigmented according to the literature [33]. The study was conducted in accordance with the Declaration of Helsinki and Good Clinical Practice, and with the approval of a Semmelweis University Regional and Institutional Committee of Science and Research Ethics (114/2012).

### RNA samples

Total RNA was extracted from the original cell suspension and from the tissue samples (subcutaneous primary tumors and metastases) at the time of evaluation, using NucleoSpin RNAII total RNA isolation kit (MACHEREY-NAGEL GmbH, Düren) according to the manufacturer's protocol.

The quality and integrity of total RNA were evaluated on 2100 Bio analyzer (Agilent Technologies, Palo Alto, CA) and the same samples were divided into individual aliquots for gene expression analysis on two different microarray platforms and for the TaqMan Gene Expression Assay based real-time PCR analysis.

### Agilent mouse oligo microarray analysis

The Agilent Mouse Oligo Microarray is comprised of 22,575 (60-mer) oligonucleotide probes representing 20,000 mouse genes and transcripts in the mouse genome. Probe labeling and hybridization were carried out following the manufacturer‘s specified protocols. Briefly, amplification and labeling of 5 μg of total RNA were performed using Cy5 for healthy control skin sample RNA and Cy3 for newborn subcutaneous tumor sample RNA. Hybridization was performed for 16 hrs at 60°C and arrays were scanned on an Agilent DNA microarray scanner. Following the manufacturer's protocol, Agilent's Stabilization and Drying Solution (#5185-5979) was used to protect against the ozone-induced degradation of cyanine dyes on microarray slides during hybridization and processing steps. Images were analyzed and data were extracted, background was subtracted and normalized using the standard procedures of Agilent Feature Extraction Software A.7.5.1. Arrays were analyzed. Linear & LOWESS, the default normalization method in the Agilent Feature Extraction Software A.7.5.1, were applied for normalizing Agilent microarrays. The method performs a linear normalization across the entire range of data, followed by a non-linear normalization (LOWESS) to the linearized data set.

### cDNA synthesis

One μg of total RNA was reverse transcribed using oligo(dT)/random primer (2.5 μM) and MMLV reverse transcriptase (200 unit/μl, Finnzyme® Espoo, Finland). The reaction mixture was incubated at 37°C for 50 min, heated at 85°C for 10 min.

### PCR

The primers were designed by ArrayDesigner software. DNA amplifications were performed using *AmpliTaq Gold® 360* PCR *Master Mix (Thermo Fisher Scientific Inc.)* and Mastercycler gradient thermal cycler supplied by Eppendorf at 35 cycles of denaturation at 95 ^°^C for 1 min, primer annealing at 59 ^°^C for 1 min, chain elongation at 72 ^°^C for 2 min. After amplification 10 μl of PCR products were separated on 3% agarose gel and stained with ethidium bromide.

### Quantitative PCR analysis

For quantitative measurement of the expressed genes (Fam 187b, Lass5, DEAD box 60 polypeptide, Gm4262, CCL11, Pex2, CathepsinL, mouse CCL12, Ninein, human CCL8, CCR1) q-PCR reactions were used Each 25 μl reaction mixture contained 12.5 μl of 2X iQ SYBR® Green Supermix (Bio-Rad), 0.5 μl of each primer for final concentration of 200 nM and 11.5 μl of the diluted cDNA. The used primers are presented in a separate table (Table 2). Cycling conditions comprised 3 min of iTaq™ DNA polymerase activation at 95°C, 40 cycles at 95°C for 30 sec, at 55°C for 30 sec and at 72°C for 1 min. Starting quantities were defined on the basis of standard fivefold dilution series (1X-625X) carried out with control cDNA of mouse B16 and human K562 cell lines. Relative expression of the examined genes was determined by normalizing starting quantities to those of the housekeeping genes beta-2 microglobulin or beta actin from the same cDNA sample.

**Table 2:** Primer sets used

Mouse specific primers			
Gene Name	Ref. Seq. Number	Sense primer	Antisense primer
Fam187b	AK028081	TCTTCCAGCCTGCCACTTAC	TGACTCCTCTTCTTGCCGC
Lass 5	NM_028015	AAGCAACTGGACTGGAGTGT	GAGAGTGGCTGATACGGATAGT
DEADbox60polypeptide	NM_001081215	GAGTACAATACGCAAGTGACAGA	TTGGAACTCCTGGACTAAGCAA
Gm4262,	AK050866	AGGAAGAACACCACAGACCAA	TATGACTACTTGTGCTCTGCCT
CCL11	NM_011330	GGCTTCATGTAGTTCAGATGG	GCTGCTATTATCCTCAGTTACTCC
Pex 2	NM_008994	TGACAGACCGCCTCCTTGG	ATGACCAGCAGCACCAGTAA
CathepsinL	NM_009984	CGCAAGCCATCCGTCTCT	GTGTCCATAAGTCCTCATTACCG
CCL12	NM_011331	CTGGTTCCTAGCTCCCCTAGC	TGGCTGCTTGTGATTCTCCT
Ninein	NM_008697	AGAAGCGAGTCAGCGAGC	CTCACCTTCTCCTCAGTCCAG
B2M	NM_009735	AACACAGTTCCACCCGCC	GTAGACGGTCTTGGGCTCG
**Human specific Primers**			
Gene Name	Ref. Seq. Number	Senseprimer	Antisense primer
Beta Actin	NM 001101	TCTGGCACCACACCTTCTAC	CTCCTTAATGTCACGCACGATTTC
CCL8	NM 005623	TTCTGTGCCTGCTGCTCATG	TTGGATGTTGGTGATTCTTGTGTAG
CCR1	NM 001295.2	GGACTATGACACGACCACAGA	GCCAGGTTCAGGAGGTAGATG
CCR2	NM 001123396	AACGAGAGCGGTGAAGAAGTC	GGTTGAGCAGGTAAATGTCAGTC
CCR5	NM 000579.3	CTGCCTCCGCTCTACTCAC	TGAAGAAGATTCCAGAGAAGAAGC

### Immunohistochemistry

Formalin fixed paraffin embedded sections of tumors were cut at a thickness of 2 μm. Sections were deparaffinated and to remove the melanin pigments we used potassium permanganate (KMnO4) with oxalic acid depigmentation protocol. Citrate buffer (pH 6) was used for microwave assisted antigen retrieval. After endogenous peroxidase quenching the sections were incubated overnight with mouse monoclonal CCL8 primary antibody in a dilution of 1:50 (Santa Cruz). Following extensive washings with PBS, sections were incubated for 1 h in the secondary biotinylated antibody, then the slides were developed by avidin-biotin peroxidase followed by incubation with diaminobenzidine or NovaRED™ Substrate (Vector Burlingame, CA). The negative control was prepared by omitting the primary antibody.

### Cell lines

The A2058 melanoma cell line was provided by LA Liotta (NCI, Bethesda, MD). HT168 and HT168M1 lines are derivatives of A2058. HT199 was developed in the 1st Institute of Pathology and Experimental Cancer Research (Semmelweis University, Budapest, Hungary). WM983B and WM983A were gifts from M. Herlyn (Wistar Institute, Philadelphia, PA). Normal human melanocytes (C-12403), keratinocytes (C-12003) and dermal fibroblasts (C-12360) were derived from Promo Cell. Cells were maintained in RPMI 1640 medium supplemented with 5% fetal bovine serum (Sigma, St. Louis, MD) at 37°C in 5% CO_2_ atmosphere.

### Cell viability test

Cells (HT199, HT168, WM983A, fibroblast and melanocyte) were placed in 96-well tissue culture plates (6×10^3^/well) in RPMI 1640 medium containing 5% FCS. After overnight incubation at 37°C, adherent cells were treated with CCL8 (R&D Systems, Minneapolis, MN, USA) (at final concentration of 500 pg/ml, 1 ng/ml) for 12 hrs. At the end of the incubation period, a colorimetric assay (MTT test) was performed. Absorbance at 570 nm was measured with Bio-Tek Microplate Reader (Merck).

### Transmembrane migration assay

Human melanoma (HT168, WM983A), fibroblast and melanocyte cell migration was measured using a real time system (Roche xCELLigence). Cells were seeded at 2×10^5^/ml in 100 μl of RPMI 1640 medium. The first round of cells were treated with 1 and 10 ng/ml of exogenous CCL8 (R&D Systems) in the upper chamber with 8 μm pore size and the second round in the lower chamber as a chemoattractant. The control cells were treated with equal volume of PBS. Cell migration was monitored every 5 min by CIM plate 16 for a period of 0-23 hrs.

### ELISA

The human CCL8 ELISA kit (R&D Systems, according to the manufacturer's protocol) was used to detect CCL8 in the serum of melanoma patients. Duplicated samples were analyzed using Bio-Tek Microplate Reader (Merck). Positive control wells were treated with recombinant CCL8 peptide.

### miRNA assay

Digital quantification of microRNA was calculated using the nCounter System from nanoString Technologies.

### Statistics

Results of quantitative PCR were analyzed with Stat Soft Statistica 11 software using unpaired t test. Cell viability and migration assays were analyzed using ANOVA and Scheffe post hoc test. Values of P ≤0.05 were considered significant.

## List of abbreviations

EMT: Epithelial-mesenchymal transition
PCR: Polymerase chain reaction
SCID: Severe combined immunodeficiency (mouse)
